# Reporting of Research Ethics in Studies Focusing on Foot Health in Patients with Rheumatoid Arthritis – A Systematic Review

**DOI:** 10.1177/15562646211047654

**Published:** 2021-10-14

**Authors:** Minna Stolt, Emilia Kielo-Viljamaa, Anne-Marie Laitinen, Riitta Suhonen, Helena Leino-Kilpi

**Affiliations:** 1 8058 University of Turku, Turku (Finland); 2 60652 Turku University Hospital, Turku (Finland); 3 96892 City of Turku, Turku (Finland)

**Keywords:** research ethics, foot health, rheumatoid arthritis, informed consent

## Abstract

Research ethics is a fundamental part of the entire research. Patients with rheumatoid arthritis are sensitive group of research participants because their long-term health problems cause significant changes in their foot health. In foot health research, data are usually collected through a clinical assessment of the foot or questionnaires. However, there is limited evidence of the reported research ethics of empirical studies on foot health in patients with rheumatoid arthritis. Therefore this review aimed to analyze the reported research ethics of peer-reviewed empirical studies focusing on foot health in patients with rheumatoid arthritis as research participants. This systematic review used the Medline/PubMed, CINAHL, and Embase databases. A total of 1,653 records were identified, and 32 articles were included in the final analysis. Reporting research ethics in studies of patients with rheumatoid arthritis is fragmented, focusing predominantly on ethical approval and informed consent and lacking a broader discussion about research ethics.

## Introduction

Research ethics is a prerequisite for robust science in any field and a fundamental part of every step in the research process. Traditionally, ethical principles of scientific research concern respect for individuals, beneficence, justice, and respect for communities (Beauchamp & Childress, 2013). Currently, these principles are strengthened by universally agreed requirements and declarations, such as the Universal Declaration on Bioethics and Human Rights (UNESCO, 2005), and the European Code of Conduct for Research Integrity (Allea, 2017). Moreover, the International Committee of Medical Journal Editors (ICMJE, 2019) has published uniform requirements for scientific publications. Ethical requirements in the field of foot health research are the same as in other fields; however, a systematic description of the reporting of research ethics in foot health research is lacking. In this study, foot health research is a scientific empirical research that focuses on the foot and ankle in patients with rheumatoid arthritis.

The Declaration of Helsinki, drawn up by the World Medical Association (originally 1964, last updated in 2013) is a cornerstone document for research ethics and states the ethical principles for medical research involving human research participants (WMA, 2013). Its fundamental principle is respect for the individual, individuals’ right to self-determination, and their right to make informed decisions. These rights relate not only to participation in research but also to the whole research process, thus giving an individual the right to withdraw from the study without individual consequences. When designing and planning the research, researchers need to clearly state in the research plan how ethical considerations and the principles of the Declaration of Helsinki are addressed in every phase of the study. This plan should be exposed to external evaluation by a research ethics committee before the study begins (WMA, 2013). Privacy has been seen as an increasingly important ethical principle in clinical research (Nurmi et al., 2019). Along with the Declaration of Helsinki, at the European level, the General Data Protection Regulation (GDPR, European Union, 2016) stipulates the privacy of research data and good practice in handling sensitive information. In addition, country-specific ethical principles support the guidance of scientific research.

Institutional review board (IRB) is independent body consisting of qualified members (WMA, 2013). An IRB might be found within a research institution or operate on a regional or national basis (WHO, 2009). The main task of this board is to evaluate compliance with internationally and nationally accepted ethical guidelines and the laws and regulations of the particular countries in which the study will take place (WHO, 2009; WMA, 2013). The main issues under evaluation are with respect to the Declaration of Helsinki and the protection of human participants (WHO, 2009). These points are evaluated based on described participant selection procedures, balance between benefits and harms, and informed consent procedures (WMA, 2013).

Besides ethical approval, permission to conduct the study is required. The application for permission depends on the organization's practices and policies (Emanuel et al., 2008; Taljaard et al., 2014). When applying for permission, the researcher must indicate how ethical guidelines will be followed in the study and what requirements of the study involve the organization in question. In health research, these requirements could involve, for example, naming a contact person (gatekeeper), infrastructure for data collection, or information technology support, for example, when dealing with patient records (Gallo et al., 2012). In some countries, permission to conduct research at a site can be obtained from the IRB at the same time as the ethics of the study are evaluated (Bracken-Roche et al., 2017; O'Mathúna, 2015; Wu et al., 2019).

Informed consent is fundamental to research ethics and is addressed in worldwide ethics guidelines (Belmont Report, 1978; Nuremberg Code, 1947). Informed consent must be obtained before enrolling any study participants in all types of research involving human subjects (Emanuel et al., 2008), and obtaining it involves a process (Kadam, 2017). The process starts by explaining the research procedures, risks, benefits, and rights of the participants (Hammond, 2016). Potential participants usually receive written and oral information about the purpose of the study, data collection, and ethical considerations (Manti & Licari, 2018). Along with this information, the potential participants are provided with an opportunity to ask questions and discuss the study with the researcher to clarify unclear issues or gain more information (Kadam, 2017). Throughout the process, a researcher needs to be aware of the participants’ ability to understand and take in information, thus being competent to provide consent (Beauchamp & Childress, 2013). Finally, the potential participant decides whether to take part or decline participation in the study (Kadam, 2017). Informed consent enables the participants to make rational and voluntary decisions about participation in the study and addresses the understanding of the research process and its risks and benefits (Beauchamp & Childress, 2013). The process of consenting is continuous and is not restricted only to initial consent; the participant has the right to withdraw from the study at any time without providing any explanation or without any effects on their healthcare provision (Emanuel et al., 2008). Informed consent can be provided in written or oral form, and when necessary, may involve parental consent/permission (when investigating children) or assent (child's affirmative agreement to participate when underage, Emanuel et al., 2008), or a surrogate decision-maker responding together with the participant, as is found occasionally in patients with memory disorders (Howe, 2012).

In general, ethics-related research on foot health is scarce. Despite this limited attention, inspecting foot health may pose several ethical issues that need to be addressed. In foot health research, the data are collected from patients, usually through a clinical assessment of the foot (Stolt et al., 2017), where participants need to show their feet to the clinician or nurse. However, revealing one's feet is seldom considered an ethical issue. Some participants who have, for example, severe foot health problems such as those with rheumatoid arthritis, or those who feel the appearance of their feet may not correspond with esthetic expectations, may be reluctant to show their feet or, if they do show their feet, they might feel embarrassed or ashamed. In foot health research, participants’ autonomy, beneficence, and non-maleficence (Helfand, 2006), as well as privacy, integrity, and respect are issues to be considered when facing the patient (Helfand, 1993). An individual approach is fundamental in facing, caring for, and researching people with foot problems because patients’ feet are unique, and their needs are personal (Stolt, 2019). Moreover, professional responsibilities to do good and to avoid harm are key issues in providing ethical high-quality care (Helfand, 1993). International ethical guidelines seem to be lacking in the field of foot health research. However, in rheumatology, along with discussions about ethics in caring for patients with rheumatic diseases (Mckeown, 2015), the European League Against Rheumatism (EULAR) have incorporated research ethics in their recommendations for the reporting of clinical trial extension studies in rheumatology (Buch et al., 2015) and the use of big data in rheumatic and musculoskeletal diseases (Gossec et al., 2020).

Previous reviews have identified inadequate reporting of research ethics, for example, while conducting studies in nursing education (Vierula et al., 2016), with clusters (Taljaard et al., 2011), stepped wedge randomized trials (Taljaard et al., 2017), dental care of older people (Mukherjee et al., 2017), and long-term care settings (Suhonen et al., 2013). The main deficits focus on reporting informed consent (Mukherjee et al., 2017; Suhonen et al., 2013; Taljaard et al., 2011; Taljaard et al., 2017; Trung et al., 2017; Vierula et al., 2016), research ethics review (Taljaard et al., 2017; Trung et al., 2017; Vierula et al., 2016), and access to a research site when studying a vulnerable patient group (Gehlert & Mozersky, 2018). Research on rheumatoid arthritis is expected to grow along with the development of technologies and advanced research methods (Cassotta et al., 2020), thus obligating researchers to commit to ethical standards and good scientific practice. However, a systematic review of ethics in foot health research among patients with rheumatoid arthritis is lacking.

## Aim

The aim of this study was to analyze the reported research ethics of peer-reviewed empirical studies focusing on foot health in patients with rheumatoid arthritis as research participants. The ultimate goals were to provide information about the quality of reported research ethics, enhance ethical discussions in the field of foot health research, and promote a high ethical standard in foot health research.

The following questions were set:

- What was the justification to conduct the study reported?
- How were ethical approval and permission for data collection obtained?
- How was informed consent obtained?

## Methods

A systematic review was conducted to synthesize information on research ethics from empirical studies that focus on foot health in patients with rheumatoid arthritis. The present study followed good scientific practices in every phase (Allea, 2017). The Preferred Reporting Items for Systematic Reviews and Meta-Analyses (Page et al., 2021) were used to guide this review.

### Literature Search

**Figure 1.**
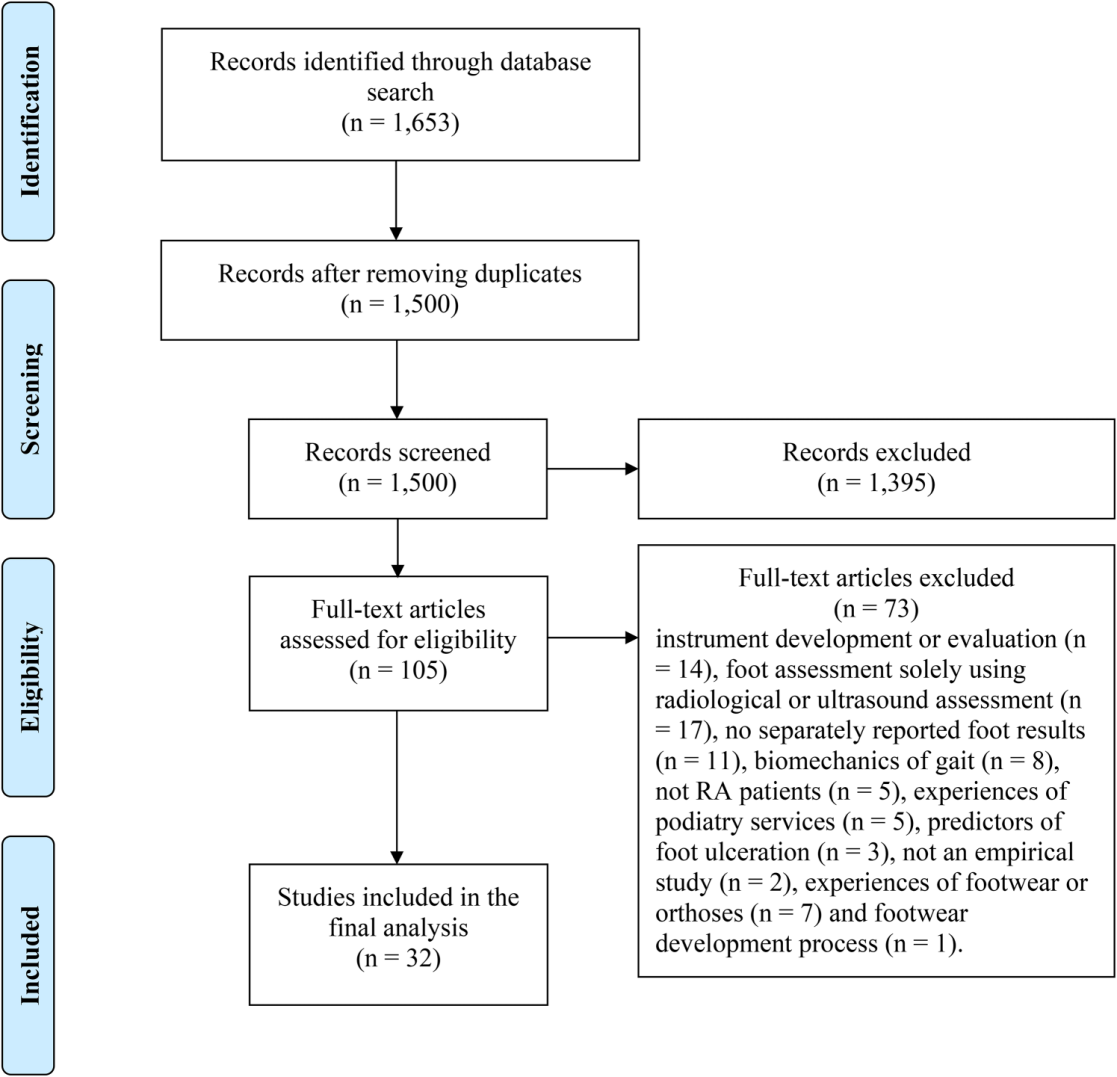
Flow chart of the literature search and retrieval process.

The articles were identified through a systematic literature search of the MEDLINE (PubMed), CINAHL, and Embase databases (Figure 1). The search was conducted on each database from the earliest available to March 31, 2021 and was limited to the English language and title/abstract level. The search terms included the following: rheumatoid arthritis AND (foot OR feet) AND (problem* OR disorder* OR complaint* OR patholog* OR deformit* OR disabilit* OR condition*). The search returned 1,653 records: 631 from Medline/PubMed, 58 from CINAHL, and 964 from Embase.

### Inclusion and Exclusion Criteria and Data Retrieval

Predetermined inclusion and exclusion criteria were applied throughout the retrieval process (Table 1). The data retrieval process consisted of three phases, where two researchers (MS, EK-V) worked independently and discussed which records were to be included in the next phase. First, in the screening phase, after removing duplicates (n = 153), all the remaining 1,500 records were screened on the title and abstract levels against the inclusion and exclusion criteria. A total of 1,395 records were excluded, resulting in 105 records for full-text analysis. Next, the full-texts were inspected, leading to the exclusion of 73 records because they reported instrument development or evaluation (n  =  14), foot assessment using solely radiological or ultrasound assessment (n  =  17), foot health results were not reported separately (n  =  11), the focus was on the biomechanics of gait (n  =  8), patients with rheumatoid arthritis were not study subjects (n  =  5), described experiences of podiatry services (n  =  5), predictors of foot ulceration (n  =  3), experiences of footwear or orthoses (n  =  7), footwear development process (n  =  1), or were not empirical studies (n  =  2). Finally, 32 records were included in the review and final analysis stages.

**Table 1.** Inclusion and Exclusion Criteria.

Inclusion criteria	empirical articles foot examinations done with clinical assessment, palpation, and/or visual observation patients with rheumatoid arthritis as study participants results providing insight into foot health (skin, nail, structure, foot pain, footwear, foot ulcers)
Exclusion criteria	surgical procedures (e.g., arthrodesis, arthroplasty) instrument validation radiographic imaging foot pressure analysis or gait and kinematics evaluation of foot problems solely using imaging or ultrasound techniques care and treatment of rheumatoid arthritis with drugs, surgery or footwear and orthosis other rheumatoid diseases

### Data Extraction and Analysis of the Studies

The following descriptive information were gathered from each study in a separate spreadsheet: author, year of publication, country of origin, main topic under investigation, design, data collection method, setting, and number of participants. Related to research ethics a separate table was formed and following aspects were gathered from the studies: how ethical approval was obtained and from which organization or body, the method of requesting informed consent and the form (oral or written), and the person who gave informed consent. Further, the process of informed consent was examined following the procedure in the Belmont report (Belmont Report, 1978), which consists of three phases: (1) providing information about the study, (2) possibility of discussing the study and opportunity to ask questions, and (3) informed consent to participate. Finally, all possible statements or considerations related to research ethics described in any part of the article were examined. The data were analysed with content analysis and quantification.

## Results

### Description of the Studies

Most of the studies (n  =  18, 56%) were conducted in Europe, of which the majority (n  =  8) were in the UK, followed by the Netherlands (n  =  5), Spain (n  =  2), and one in Poland, Portugal, and Sweden. Five studies were conducted in New Zealand, two in the United States, and one in Australia, Brazil, Colombia, Iran, Japan, Korea, and Turkey (Table 2).

**Table 2.** Characteristics of the Studies (n = 32).

Author, year, country	Aim	n	Mean age (range or SD)	Design	Setting	Data collection method
Aleixo et al., 2018 Portugal	to comparise of rheumatoid arthritis post-menopausal women with pathological involvement of the lower limb joints and age-matched healthy post-menopausal women in regard to the dynamic joint stiffness of the ankle during the stance phase of gait.	36	64.3 (8.4)	NR	institute of rheumatology	gait analysis
Bagherzadeh Cham et al., 2014, Iran	to evaluate the effect of heel-to-toe rocker shoe on pain, disability, and activity limitation in RA patients immediately, 7 and 30 days after their first visit.	17	47.16 (29-60)	clinical trial	NR	foot deformities, foot/ankle range of motion
Borman et al., 2012, Turkey	to evaluate the foot involvement in regard to symptoms, type and frequency of deformities, location, radiological changes, and foot care in a group of RA patients.	100	52.2 (27-79)	NR	outpatient clinic	clinical data, physical examination, questionnaire
Brenton-Rule et al., 2014, New Zealand	to evaluate the effect of open-back and closed-back sandals, in relation to postural stability, in women with established RA.	20	67.7 (44-84)	exploratory study	outpatient clinic	postural stability, current disease activity
Brenton-Rule et al., 2016, New Zealand	to determine the foot and ankle characteristics associated with falls in people with RA.	201	64.7 (11)	cross-sectional study	outpatient clinic	foot and ankle characteristics
Cameron-Fiddes and Santos, 2013, UK	to investigate the effects of off the shelf foot orthoses on outcomes of swollen and tender joints, and pain, in patients with early rheumatoid arthritis.	35	52.4 (26-80)	within subject controlled study	rheumatic disease unit	biomechanical assessment
Cho et al., 2009, Korea	to evaluate the effects of different types of foot orthoses combined with specialized orthotic shoes in rheumatoid arthritis patients with foot involvement.	42	48.7 (11.6)	randomized controlled trial	outpatient clinic	clinical assessment, foot pain and function
Dahmen et al., 2014, the Netherlands	to investigate the use and effects of therapeutic footwear.	114	60 (48-67)	cross-sectional, observational study	arthritis foot clinic	foot characteristics, footwear
Dahmen et al., 2020, the Netherlands	to explore the associations between body mass index (BMI) and measures of foot health in patients with RA and foot complaints	230	58 (13)	cross-sectional study	outpatient clinic	clinical exam, plantar pressure measurements, questionnaires
de Andrade et al., 2018, Brazil	to analyze the prevalence of foot involvement in a sample of Brazilian RA patients and to explore the influence of disease variables such as inflammatory activity, serological, and epidemiological profile in this type of involvement.	200	55.2 (20–76)	cross-sectional study	outpatient clinic	questionnaire, clinical data
Douglas et al., 2006, UK	to determine the prevalence and characteristics of dermatological disorders in contemporary patients with rheumatoid arthritis and compare this with a group of patients with non-inflammatory rheumatic conditions.	205	61.52 (12.88)	cross-sectional, observational study	outpatient clinics	dermatological examination
Fransen and Edmonds, 1997, Australia	To assess the effectiveness of off-the shelf orthopedic footwear for people with rheumatoid arthritis (RA) reporting chronic foot pain, in terms of self-reported pain and physical function, as well as objectively measured gait variables using an electric footswitch walkway	30	59.1 (14.2)	randomized controlled trial	public hospital	assessment of gait, pain, physical function
González-Fernández et al., 2016, Spain	to investigate the presence of biomechanical abnormalities and US-detected inflammation and damage in low disease or remission status RA patients as compared to healthy subjects, both with foot complaints.	117	57.1 (23-78)	cross-sectional study	hospital, rheumatology department	podiatric assessment
Grondal et al., 2008, Sweden	to investigate the distribution of joint involvement in RA patients today, with special attention given to the feet and subjective walking ability.	1000	60 (19-88)	cross-sectional study	outpatient clinic	questionnaire
Kerry et al., 1994, UK	to assess the incidence of both forefoot and hindfoot problems and their importance as a cause of disability.	100	female: 59 (26-80), male: 56 (35-81)	NR	NR	foot clinical assessment
Konings-Pijnappels et al., 2019, the Netherlands	To investigate the association of plantar pressure with disease activity and deformity in the forefoot in patients with rheumatoid arthritis and forefoot symptoms.	172	57.9 (12.9)	cross-sectional study	foot-care clinic in outpatient center	planter pressure tests, forefoot deformity
Lohkamp et al., 2006, UK	to gather data about the prevalence of foot pain and information about the impact of foot problems on disability in patients with early diagnosed RA.	185	53 (16-84)	NR	outpatient clinic	questionnaire
Michelson et al., 1994, USA	to examine the prevalence of foot and ankle problems in 99 patients with clinically proven rheumatoid arthritis.	99	58.4 (1.2)	cross-sectional study	outpatient rheumatology clinic	physical exam
Mochizuki et al., 2019, Japan	to investigate the relationship of callosities of the forefoot with foot deformity, the Health Assessment Questionnaire Disability Index and modified total Sharp score in patients with rheumatoid arthritis.	202	65.9 (13.3)	NR	outpatient clinic	dermatological assessment
Morpeth et al., 2016, New Zealand	to evaluate the relationship between fear of falling and foot pain, walking velocity and foot impairment and disability in women with established RA.	21	66 (10)	NR	podiatric rheumatology outpatient clinic	clinical tests, questionnaires, walk test
O'Connell et al., 1998, USA	to evaluate how painful metatarsal arthritis affects foot and ankle mechanics and mobility	10	54	NR	NR	foot function during gait
Park and Craxford, 1981, UK	to assess clinical impression that there was a moderately high level of disapproval with various aspects of bespoke footwear	71	NR	NR	hospital departments	questionnaire
Reinoso-Cobo et al., 2020, Spain	to identify foot health factors related to the quality of life in patients with rheumatoid arthritis (RA).	293	median age 59	cross-sectional study	hospital outpatient clinics	clinical assessment
Rojas-Villarraga et al., 2009, Colombia	to determine the main foot alterations in RA patients and to measure the impact of foot impairment on global QOL based on validated scales and their relationship to disease activity.	95	52.5 (12.4)	cross-sectional study	rheumatology unit patients	foot examination, questionnaires
Rome et al., 2009, New Zealand	to identify the nature of foot problems experienced by patients attending rheumatology outpatient clinics at Counties Manukau District Health Board and to ascertain the availability of podiatric services for these patients.	100	51-64	clinical audit	rheumatology outpatient clinics	foot and ankle assessment
Siddle et al., 2012, UK	to describe the clinical characteristics of foot ulceration in patients with rheumatoid arthritis (RA).	32	72 (45-85)	NR	rheumatology foot health clinic	clinical examination
Silvester et al., 2010, New Zealand	to identify footwear style, footwear characteristics, and key factors influencing footwear choice using objective footwear assessment tools.	80	60 (51-70)	NR	rheumatology outpatient services	clinical data, function, foot impairment, disability
van der Leeden et al., 2006, the Netherlands	To assess (i) the relationship between forefoot joint damage and foot function (expressed as gait and pressure parameters), (ii) the relationship between foot function and pain, and (iii) the relationship between foot function and disability in patients with foot complaints secondary to rheumatoid arthritis (RA).	62	55.7 (13.11)	cross-sectional study	out-patient clinic for rehabilitation and rheumatology	foot function: gait analysis, disability
van der Leeden et al., 2007, the Netherlands	To assess the relationship between disease duration and foot function, foot pain and disability, in patients with foot complaints secondary to rheumatoid arthritis (RA).	62	55.7 (13.1)	cross-sectional study	outpatient clinics	foot function, disability, foot pain
Wareńczak et al., 2014, Poland	This study presents results of the lower extremity functional test with evaluation of the pain level which occurred after each task.	12	54 (9.8)	clinical trial	outpatient rehabilitation ward	lower extremities functional test, pain
Williams et al., 2007, UK	to evaluate the clinical effectiveness of the new therapeutic shoe compared with the pre-existing one.	80	NR	randomized controlled trial	local rheumatology clinic	questionnaires
Wilson et al., 2017, UK	to determine the prevalence, impact and care of foot problems in all patients with RA in one geographical area and identify factors associated with accessing foot care.	413	62.6 (13.6)	population-based cross-sectional survey	podiatry clinic	questionnaires, clinical data

NR  =  not reported, SD  =  standard deviation.

The most common focus in the original studies was on foot deformities in patients with rheumatoid arthritis (n  =  17, 53%), followed by therapeutic footwear or orthoses (n  =  9, 28%) and foot function (n  =  6, 19%). The number of participants in the study varied between 10 and 1,000. Participants were adults whose mean age in years varied between 47 and 72 years. The most common design used in the studies was a cross-sectional study design (n  =  14, 44%). The studies were predominantly conducted in outpatient clinics for patients with rheumatoid arthritis (n  =  22, 69%). In four (13%) studies, the setting was a clinic, a unit, or health care services in general for patients without any information regarding whether the patients were inpatients or outpatients.

### Reporting the Justification to Conduct the Study

In all the studies, the justification for conducting a particular study was presented. The reasons for conducting the study were either to identify knowledge gaps in previous studies, to test a new therapeutic method, or to update existing knowledge.

### Reporting of Ethical Approval and Permission for Data Collection

Ethical approval and the review body (ethics committee) were reported in most of the studies (n  =  26, 81%, Table 3). The types of ethical review boards were independent review boards located in universities (IRBs), or anonymous local ethical committees. A few studies (n  =  3, 9%) also reported reference to either international or national ethical standards. However, only one study separately reported permission to conduct the study (Cameron-Fiddes & Santos, 2013).

**Table 3.** Reported Ethical Approval and Informed Consent in the Studies (n = 32, in Alphabetical Order).

	Informed consent			Process of informed consent			
Ethical approval	Written	Given, type not reported	Consenting person	1) Giving information	2) Discussion and opportunity for questions	3) Give informed consent	Reference
x	x		x			x	Aleixo et al., 2018
x	x		x			x	de Andrade et al., 2018
x	x		x			x	Bagherzadeh Cham et al., 2014
x		x	x			x	Borman et al., 2012
x	x		x			x	Brenton-Rule et al., 2014
x	x		x	x		x	Brenton-Rule et al., 2016
x		x		x	x	x	Cameron-Fiddes and Santos, 2013
x	x		x		x	x	Cho et al., 2009
x	x		x			x	Dahmen et al., 2014
x	x		x			x	Dahmen et al., 2020;
		x				x	Douglas et al., 2006
x	x		x			x	Fransen and Edmonds, 1997
x	x		x			x	González-Fernández et al., 2016
x		x				x	Grondal et al., 2008
							Kerry et al., 1994
x			x				Konings-Pijnappels et al., 2019
x	x		x			x	van der Leeden et al., 2006
x	x		x			x	van der Leeden et al., 2007
x	x		x			x	Lohkamp et al., 2006
							Michelson et al., 1994
x	x		x			x	Mochizuki et al., 2020
x	x		x			x	Morpeth et al., 2016
		x				x	O'Connell et al., 1998
							Park and Craxford, 1981
x	x		x	x	x	x	Reinoso-Cobo et al., 2020
x	x		x			x	Rojas-Villarraga et al., 2009
x				x			Rome et al., 2009
x	x		x			x	Siddle et al., 2012
x		x				x	Silvester et al., 2010
							Wareńczak et al., 2014
x		x				x	Williams et al., 2007
x	x					x	Wilson et al., 2017
**26 (81%)**	**19 (60%)**	**7** **(22%)**	**20** **(63%)**	**4** **(13%)**	**3** **(9%)**	**26** **(81%)**	**total (n, %)**

### Obtaining Informed Consent

Most studies (n  =  26, 81%) obtained informed consent (phase 3 according to the Belmont report, 1978), majority (n  =  19) of which obtained a written informed consent. However, in only a small number of studies (n  =  2, 6%) the informed consent process was reported, including providing information (phase 1 according to the Belmont report). In addition, only a few studies (n  =  3, 9%) reported a discussion about the study and the opportunity to ask questions (phase 2 according to the Belmont report) (Table 3). The persons who gave informed consent were the participants themselves, and this was reported in most (n  =  20, 63%) of the studies.

## Discussion

This review demonstrated that research ethics of empirical studies focusing on foot health in patients with rheumatoid arthritis as research participants are insufficiently discussed and described. This finding supports previous studies in other areas (Suhonen et al., 2013; Wu et al., 2019) and confirms the need to improved transparency and standards of ethical reporting (Wu et al., 2019).

Ethical approval and the body reviewing the study were reported in most of the studies. Obtaining informed consent was also reported in most of the studies, but permission from organization or hospital to conduct the study was reported only in one study; thus, it is unclear whether permission to conduct the study was granted by the organization or institution from which the data were collected. This may indicate that informed consent is more important when reporting study ethics. The discrepancy in reporting permission to conduct the study could be related to the varying approving bodies. The studies in this review were conducted in different countries, which may have different procedures for ethical approval and organizational permission to conduct a study. In some countries, ethical approval is first considered by an IRB within a hospital and, if approved, in line with internal delegation, permission to conduct the study is decided by the responsible body of the unit or sector in which it is planned to be conducted. In some countries, the same body, such as an IRB in a hospital, can provide both ethical approval and permission to conduct the study, whereas in other countries, for example, ethical approval is sought from a university ethical review board, while permission to conduct the study is obtained from a particular organization. However, the reporting of these permissions should be clear and unambiguous (Dunn & Jeste, 2001; Taljaard et al., 2014).

Reporting of the informed consent process was fragmented. Only a few studies have described the entire informed consent process, following the Belmont report (1978). Notably, there were some studies where the informed consent process was not reported at any level, although this does not automatically mean that informed consent was not obtained. However, scientific journals and publishers are increasingly asking for a statement on the ethics of the study during the submission process. In future studies, thorough reporting of research ethics is needed, particularly on issues related to informing participants of their rights. The articles identified in this review seldom reported on anonymity, confidentiality, and voluntariness, which are basic ethical requirements in data collection. This indicates that it is necessary to include a detailed description of data collection in the articles’ methodology section to increase critical thinking and ethical reasoning, particularly in studies with invasive research methods. According to the literature, organized, shorter, and more readable consent forms, simplified text, and illustrated formats may improve participants’ understanding (Kadam, 2017; Misra & Agarwal, 2020). As the foot health research is commonly conducted in clinical settings, it is important to highlight to participants what components belong to professional foot care and which to scientific foot-related research. Written informed consent was used in those studies that reported on informed consent, but no additional detailed structure or content of the informed consent was reported in the studies.

There are some limitations to be discussed before interpreting the results. This review focused on articles dealing with foot health in patients with rheumatoid arthritis. Articles related to surgical procedures, radiography, foot kinematics evaluation, and pharmacological studies were excluded because the focus was on non-invasive foot health studies. This might have limited the generalizability of the results to a wider patient population with rheumatoid arthritis or other long-term foot health conditions. However, ethical issues and requirements are similar in all patients and should be considered in every study. Finally, this review reports and synthesizes these ethical issues and the considerations that the original researchers have reported.

In summary, the reporting of research ethics in studies focusing on foot health in patients with rheumatoid arthritis is fragmented. It is predominantly focused on ethical approval and informed consent, and a wider discussion about research ethics is lacking. It is necessary to improve the level of reporting on research ethics by taking a more comprehensive perspective to include aspects of participants’ rights and research integrity. In foot health research, a high ethical standard is required across the entire research process. Research ethics should not only be seen as a prospective mechanical review of ethical issues while preparing documents for ethical approval (Dawson et al., 2019). Instead, the entire research process should be carried out in the most ethical way possible.

## Best Practices

This review provides information based on descriptions in research publications; therefore, it is not possible to analyze actions that have been taken in conducting these studies in different countries. The information produced in this review can be utilized to improve the quality of reporting, procedures in empirical research, and ethics education.

Patients with rheumatoid arthritis are a sensitive group of research participants because they have long-term health problems that restrict their autonomy and daily activities. Moreover, rheumatoid arthritis causes significant changes in the shape and appearance of the feet; thus, many patients generally consider their feet to be unaesthetic and do not feel comfortable revealing their feet to others. These are ethical issues that need to be considered during the entire research process.

## Research Agenda

Our findings highlight the need for further research on research ethics. The present study focused on analyzing the reported research ethics in scientific publications. However, in the future, it would be beneficial to explore researchers’ perceptions of issues related to reporting research ethics. This study focused only on a narrow group of patients; thus, in the future, it would be interesting to analyze how reporting of research ethics has been conducted among other patient groups.

## Educational Implications

Careful consideration of the ethical aspects of reporting is mandatory for every publication. However, the reporting of research ethics from a researcher's perspective is a balancing act between tighter word limits and cohesive reporting of results. Due to limited publication space, authors tend to trim their reporting of ethics to the most central aspects, such as ethical approval and informed consent, leaving out other important ethical perspectives. As ethical guidelines and data protection regulations have become more stringent, more detailed reporting of research ethics is needed. Scientific publishers could consider extending word limits and requiring a comprehensive description of research ethics. Open access publishing could provide a forum for this type of reporting. The Ethical Review Boards could provide education for researchers about research ethics and remind that reporting research ethics is as important as ethical approval.

## Source of Support:

This study was supported by the Turku University Hospital (funding reference 2018/13240).
